# Seasonal Distribution of Viral Pneumonia After COVID-19 and the Role of Hematological Markers in Assessing Pneumonia Severity: A Case–Control Study

**DOI:** 10.3390/tropicalmed10090268

**Published:** 2025-09-17

**Authors:** Şaban Melih Şimşek, Ayşe Elif Bayar

**Affiliations:** 1 Department of Chest Diseases, Giresun University, 28100 Giresun, Türkiye; 2 Department of Emergency Medicine, Giresun University, 28100 Giresun, Türkiye; ayse.bayar@giresun.edu.tr

**Keywords:** Influenza A/B, Neutrophil/Lymphocyte Ratio, Lymphocyte/Monocyte Ratio, Platelet/Lymphocyte Ratio, SARS-CoV-2, viral seasons

## Abstract

Background: Various studies have shown that viral pneumonia pathogens display distinct inflammatory profiles, and hematological indices, such as the Neutrophil/Lymphocyte Ratio (NLR), Lymphocyte/Monocyte Ratio (LMR), and Platelet/Lymphocyte Ratio (PLR), can serve as accessible markers of disease severity. Moreover, the seasonal distribution of respiratory viruses appears to have shifted during the COVID-19 pandemic. Methods: This retrospective case–control study was conducted on patients diagnosed with PCR-confirmed viral pneumonia in the emergency department of a tertiary care center between 1 January and 31 December 2024. The control group comprised age- and sex-matched individuals without viral pneumonia. Subjects with comorbidities or ongoing treatments potentially affecting hematological indices were excluded. Seasonal distribution of viral pathogens was recorded. Hematological and inflammatory parameters at admission—including neutrophil-to-lymphocyte ratio (NLR), lymphocyte-to-monocyte ratio (LMR), and platelet-to-lymphocyte ratio (PLR)—were evaluated. The associations between these biomarkers, Pneumonia Severity Index (PSI) scores, and hospitalization status were statistically analyzed. Results: In this study, it was determined that Influenza A/B was more common in winter (67.3%) and SARS-CoV-2 in summer (70.7%). The relationship between the Pneumonia Severity Index and hemogram parameters was examined in determining the severity of pneumonia. In SARS-CoV-2, leukocyte and neutrophil counts were positively correlated (R: 0.392, *p*: 0.003; R: 0.466, *p*: <0.001), while in Influenza A/B, lymphocyte, platelet, and monocyte counts showed a negative correlation (R: −0.402, *p*: 0.005; R: −0.331, *p*: 0.021; R: −0.327, *p*: 0.023). Correlations were found between inflammation parameters and the Pneumonia Severity Index, except for the Lymphocyte/Monocyte Ratio, between SARS-CoV-2 and Influenza A/B (*p* < 0.05). Conclusions: The seasonal distribution of viral pneumonia pathogens has been revealed following the COVID-19 pandemic. Due to differences in inflammation patterns in viral infections, different leukocyte subgroups have been suggested as biomarkers.

## 1. Introduction

Respiratory viral infections are a significant public health issue due to their prevalence, potential to cause outbreaks in communities, and frequent association with morbidity and mortality. Most viruses are transmitted through droplets and primarily cause infection in the epithelium of the respiratory tract. They can lead to morbidity and mortality ranging from simple upper respiratory tract infections to bronchiolitis, pneumonia, acute respiratory distress syndrome (ARDS), and respiratory failure. Among these agents, influenza A/B, parainfluenza virus, and respiratory syncytial virus (RSV) typically cause epidemics, while adenovirus, coronavirus, and rhinovirus cause endemic infections [1]. In particular, the seasonal distribution of viral infections has changed during the COVID-19 pandemic [2,3,4,5]. The increasing availability of pathogen-specific antiviral treatments, along with the varying incubation periods and treatment regimens for viruses, has made it crucial to determine their seasonal distribution. In recent years, numerous studies have been conducted on viral infection agents and their epidemiological characteristics, particularly with the identification of new-generation respiratory viruses causing respiratory failure [1,6].

Viral respiratory infections exhibit distinct patterns of inflammation [7]. In influenza A/B, inflammation is primarily mediated by CD4+ T lymphocytes, accompanied by neutrophils and monocytes. In the case of SARS-CoV-2, however, the inflammatory process also directly affects lung fibroblasts [7]. For these reasons, hematological inflammation markers play an essential role in the diagnosis and management of viral respiratory infections. Haemogram parameters, particularly the neutrophil/lymphocyte ratio, are used to assess the severity and course of disease. Studies have shown that elevated NLR and lymphopenia values are associated with more severe infections [8,9,10,11,12].

The World Health Organisation (WHO) has announced that the COVID-19 pandemic ended on 5 May 2023, lifting the Public Health Emergency of International Concern (PHEIC) status [13]. Current studies from the post-COVID-19 pandemic period have reported changes in the distribution of viral agents, although with limited sample sizes and mostly single-center data. Additionally, data on the relationship between different viral agents and their hematological profiles are insufficient. In seasonal transition regions, such as Turkey, evaluating the relationship between the seasonal distribution of viral pneumonia agents and disease severity during a period of full reopening post-pandemic, like 2024, would fill a significant information gap from both epidemiological and clinical perspectives.

In this study, the aim was to determine the prevalence and seasonal distribution of viral pathogens detected in patients with symptoms and findings of viral pneumonia in 2024, following the COVID-19 pandemic, and to examine the relationship between haemogram parameters and disease severity retrospectively.

Viral agents exhibit different inflammatory patterns. In this study, unlike many studies in the literature, multiple viral agents were evaluated both together and separately, and differences between them were identified through haematological parameters. Additionally, it is known that mild viral infections may not cause significant changes in haematological parameters [14]. By including patients diagnosed with viral pneumonia in the study, the effect of moderate to severe viral inflammation on haematological parameters could be evaluated more reliably.

We hypothesized that viral infections tend to be more common during the winter months. Additionally, thrombocytopenia and lymphopenia are linked to increased disease severity, often resulting in hospitalization for patients with viral infections. Increases in the neutrophil-to-lymphocyte ratio, lymphocyte-to-monocyte ratio, and platelet-to-lymphocyte ratio during viral infections are associated with worsening disease severity, which may necessitate hospitalization.

## 2. Materials and Methods

### 2.1. Design and Population

This study was designed as a retrospective case–control study. Patients aged 18 years and older who presented to the emergency department of our tertiary care hospital between 1 January 2024, and 31 December 2024, with symptoms and findings consistent with viral pneumonia, were included in the study. Data were obtained from Giresun University Training and Research Hospital, Giresun, Turkey. Following the COVID-19 pandemic, routine sputum culture and viral polymerase chain reaction (PCR) kit tests have been used at our hospital to identify the causative agent in patients with pneumonia symptoms. In this study, sputum culture was requested for all patients. However, culture results that were deemed inappropriate or contaminated were excluded from the analysis. In addition, viral polymerase chain reaction (PCR) testing was routinely performed in our hospital following the COVID-19 pandemic to identify the causative agent in patients with pneumonia symptoms. Through the Hospital Information Management System (HIMS), a list of patients who underwent viral respiratory panel testing in our hospital’s emergency department between 1 January 2024, and 31 December 2024, was obtained. Information on the patients’ recorded symptoms, physical examination findings, vital signs, laboratory results, radiological imaging, comorbidities, occupation, medical history, family history, and medication was obtained. Patients in whom the causative agent was identified as a virus were selected for the study group.

### 2.2. Inclusion and Exclusion Criteria

Patients with bacterial growth detected in sputum culture, smokers, and those with multiple viral agents detected were excluded from the study due to potential confounding factors. The use of medications or substances that could affect haemogram parameters (corticosteroids, immunosuppressants, etc.) and the presence of comorbidities (acute organ failure, chronic kidney disease, malignancies, haematological disorders, etc.) were established as exclusion criteria. Subsequently, patients without viral pathogens and those who did not meet the inclusion and exclusion criteria were excluded. Since many factors can influence haemogram parameters as confounding factors, careful screening was performed. However, while this may result in data loss during screening, we believe that it enhances the internal validity of the data obtained.

The control group was determined using the same inclusion and exclusion criteria as the study group. An equal number of individuals with similar age and gender distribution were selected from the control group. However, the control group will be evaluated separately from viral factors. This will ensure a 1:2 or 1:3 ratio between the study and control groups. In studies, it has been recommended that the case and control group numbers be distributed in a 1:2, 1:3, or 1:4 ratio to ensure that the data reflects the population in case–control studies [15,16].

### 2.3. Viral Polymerase Chain Reaction (PCR) Diagnostic Kit

In this study, the Bio-Speedy^®^ Respiratory ID-2 Kit (Bioeksen R&D Technologies Ltd., Istanbul, Turkey) PCR kit was used to detect viral pathogens in respiratory samples during the relevant period at our hospital. Samples were collected via the nasopharyngeal route. The kit demonstrated high sensitivity and specificity in detecting viral pathogens, including SARS-CoV-2, influenza A/B, RSV A/B, Adenovirus, Human Rhinovirus, and Group A Streptococcus [17]. Although this is a single-centre study, the standardised and reliable diagnostic test used provides a strong methodological advantage in this regard.

### 2.4. Pneumonia Severity Index (PSI)

In this study, cases were classified according to the viral agents detected; the seasonal distribution, disease severity, and inflammatory response of each group were examined. In terms of haemogram parameters, the control group was compared separately with subgroups divided according to viral agents. The pneumonia severity index (PSI) and the number of lung lobes with pneumonic infiltration were used to assess the severity of pneumonia-related illnesses. Standardization was planned in this way.

PSI is an index that has been shown to predict mortality in community-acquired pneumonia by Flanders et al. [18]. Kim et al. demonstrated that it can also be effectively used in viral community-acquired pneumonia [19]. This index assesses the severity of community-acquired pneumonia by considering age, gender, nursing home residence, and comorbidities.

### 2.5. Primary, Secondary Outcomes, and Potential Biases

The primary outcome of the study was to determine the frequency and seasonal distribution of viral pneumonia pathogens. The secondary outcome was to compare disease severity with haematological inflammation parameters.

The study has some potential methodological biases. These are mainly: 

The possibility of patients being excluded from or included in the study due to missing data during HBYS data screening, Errors in viral sample collection in busy clinical settings, such as emergency departments, may lead to false-positive or false-negative results. The presence of viral pathogen-related infections other than those identified by the viral respiratory pathogen screening kit, we can list these as potential sources of bias. To prevent potential biases, patients with missing data were excluded. Objective scales, such as the PSI, were used. Patients with symptoms and findings consistent with viral pneumonia but no detectable viral pathogen were excluded.

Patients with viral pathogen detection but inconsistent viral pneumonia symptoms and findings were excluded. This was done to eliminate false positives and negatives. Classification based on viral agents was performed, aiming for a distribution ratio of approximately 1:2 or 1:3 between the study and control groups. This method was employed to minimize bias in the selection of the control group.

Permission for the study was obtained from the Giresun Training and Education Hospital Scientific Research Studies Ethics Committee (14.05.2025/01).

### 2.6. Statistical Analysis

Statistical analyses will be performed using the IBM SPSS Statistics 29.0 (IBM SPSS Statistics for Windows, Version 25.0. IBM Corp., Armonk, NY, USA) software package. The normality of the data will be tested using the Kolmogorov–Smirnov and Shapiro–Wilk tests. Descriptive statistics for numerical data will be presented as mean ± standard deviation (SD) if the data are normally distributed, and as median (min-max) if they are not normally distributed. Descriptive statistics for categorical variables will be presented as frequency and percentage (%). When comparing numerical data between two independent groups, the Student *t*-test is used for normally distributed data, and the Mann–Whitney U test is used for non-normally distributed data. Correlations between variables were assessed using Pearson’s correlation coefficient for normally distributed data and Spearman’s rank correlation coefficient for non-normally distributed data. Regression analyses will be used to determine the cause-and-effect relationship between the data. The statistical significance level will be considered as *p* < 0.05.

The reporting of this study was prepared per the recommendations of the “Strengthening the Reporting of Observational Studies in Epidemiology (STROBE)” guideline [20].

## 3. Results

**Figure 1:**
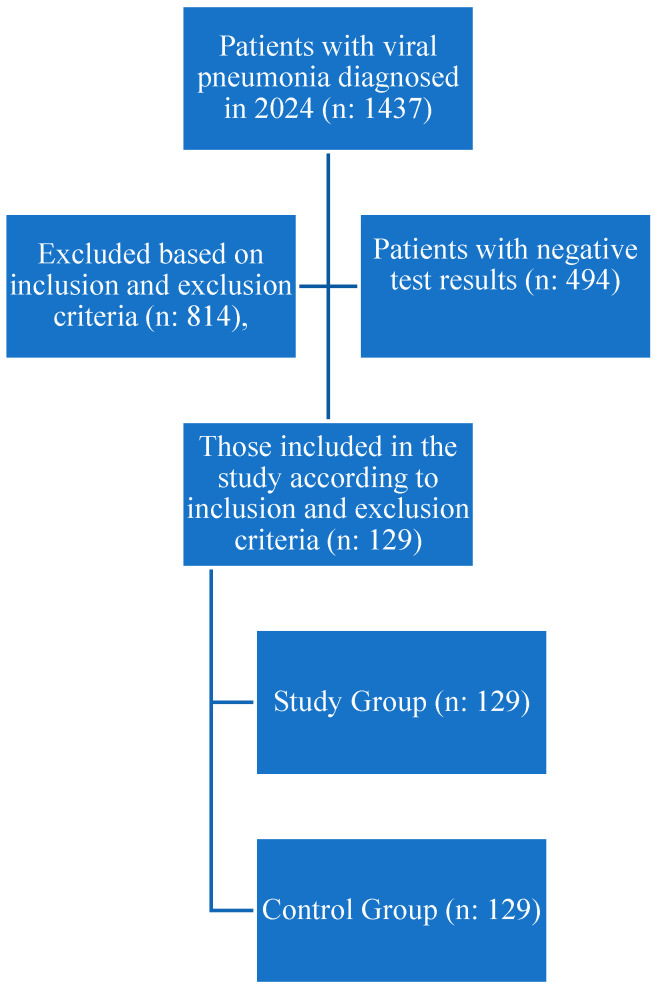
Flow Chart.

In the current study, the hospital database was searched, and it was found that 129 patients who met the inclusion and exclusion criteria and were diagnosed with viral pathogens were among 1437 patients who visited the emergency department in 2024 (Figure 1). A control group of 129 participants was also included, matching the patient group in terms of age and gender and meeting the inclusion and exclusion criteria.

**Figure 2:**
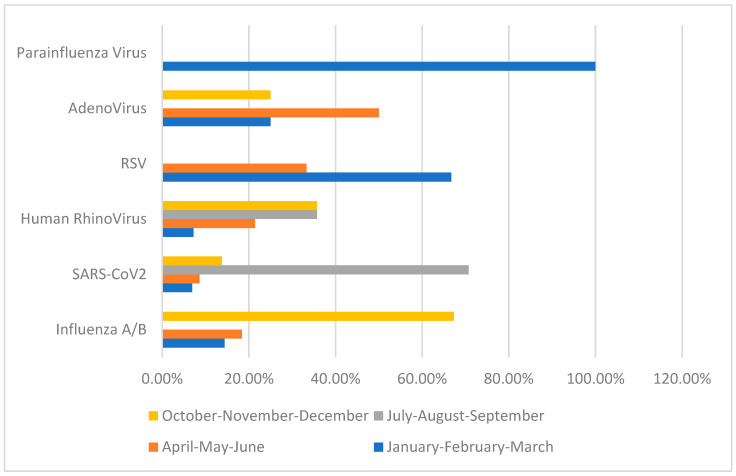
Seasonal Disturbance of Viral Pathogens.

Influenza A/B was detected in 38% (n: 49) of patients, SARS-CoV-2 in 45% (n: 58), and rhinovirus in 10.9% (n: 14) (Table 1). SARS-CoV-2 was predominantly observed in July–August–September (71.9%), while influenza A was predominantly detected in October–November–December (67.3%) (Table 1, Figure 2). Of the patients diagnosed with Influenza A/B and SARS-CoV-2, 28.6% and 24.1%, respectively, were hospitalized for treatment (Table 1).

The haemogram parameters of patients with SARS-CoV-2 and influenza A/B pneumonia were compared separately with those of the control group using a Student *t*-test. Statistically significant differences were observed in all parameters except platelet and leukocyte counts for SARS-CoV-2 patients. Significant decreases were observed in lymphocyte, monocyte, and eosinophil counts and hemoglobin levels (*p* < 0.001, *p*: 0.012, *p* < 0.001, *p* < 0.001). In contrast, neutrophil counts, NLR, LMR, and PLR showed increases (*p* < 0.001, *p* < 0.001, *p* < 0.001, *p* < 0.001, respectively). Statistically significant differences were observed in all parameters for patients with Influenza A/B. Significant decreases were observed in LMR, leukocyte, lymphocyte, monocyte, and eosinophil counts and hemoglobin levels (*p* < 0.001, *p*: 0.019, *p*: 0.012, *p* < 0.001, *p* < 0.001, respectively). In contrast, neutrophil counts, NLR, and PLR showed increases (*p*: 0.016, *p* < 0.001, *p* < 0.001, respectively) (Table 2).

No analysis was performed due to the insufficient number of patients to establish statistical significance for viruses other than SARS-CoV-2 and influenza A/B.

Haemogram parameters were examined with the Mann–Whitney test in patients diagnosed with influenza A/B and SARS-CoV-2 who were treated either as outpatients or admitted to the hospital. A positive statistical significance was found between the neutrophil/lymphocyte ratio and hospital admissions in influenza A/B and SARS-CoV-2 (*p*: 0.017, *p*: 0.00, respectively). However, in patients with influenza A, a negative correlation was found between lymphocyte (*p* < 0.001), monocyte (*p*: 0.022), and platelet counts (*p* = 0.01) and hospitalization [21,22]. In SARS-CoV-2, positive correlations were found between leukocyte (*p*: 0.004) and neutrophil (*p*: 0.00) counts and hospital stays, while a negative correlation was observed between eosinophil counts (*p*: 0.001) and hospital stays.

**Figure 3:**
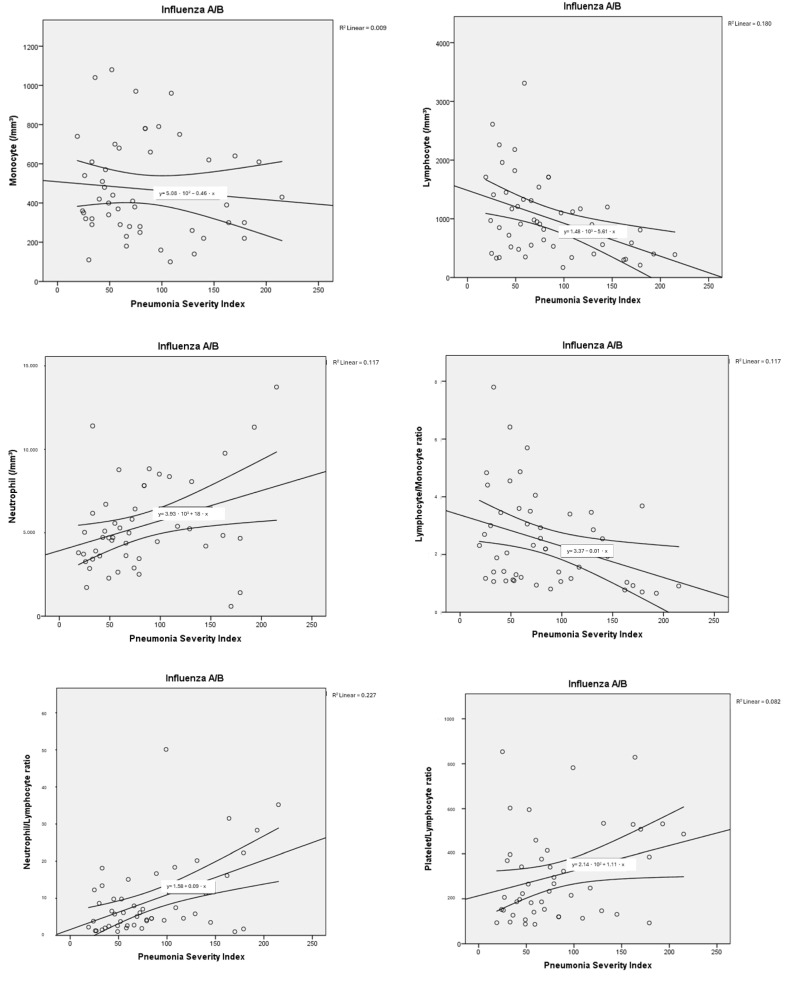

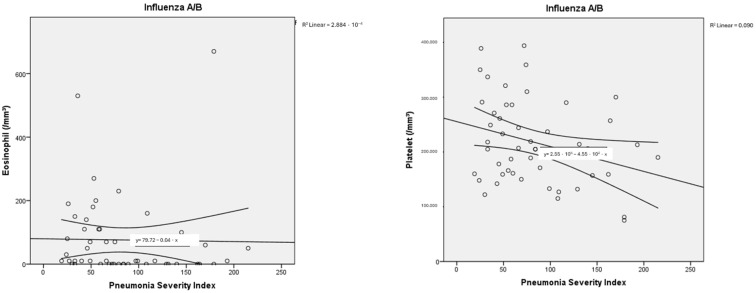
Correlation dot charts between the Pneumonia Severity Index and haematological parameters in viral pneumonia associated with influenza A/B.

**Figure 4:**
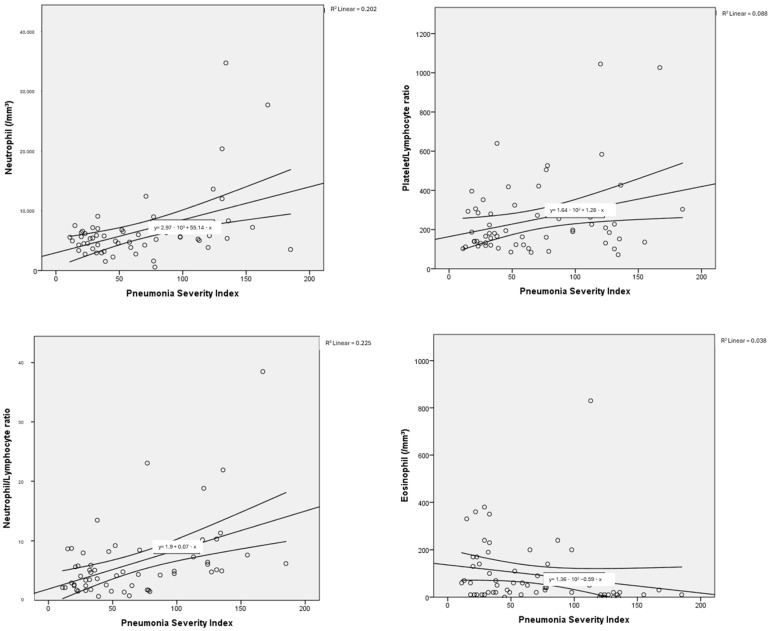

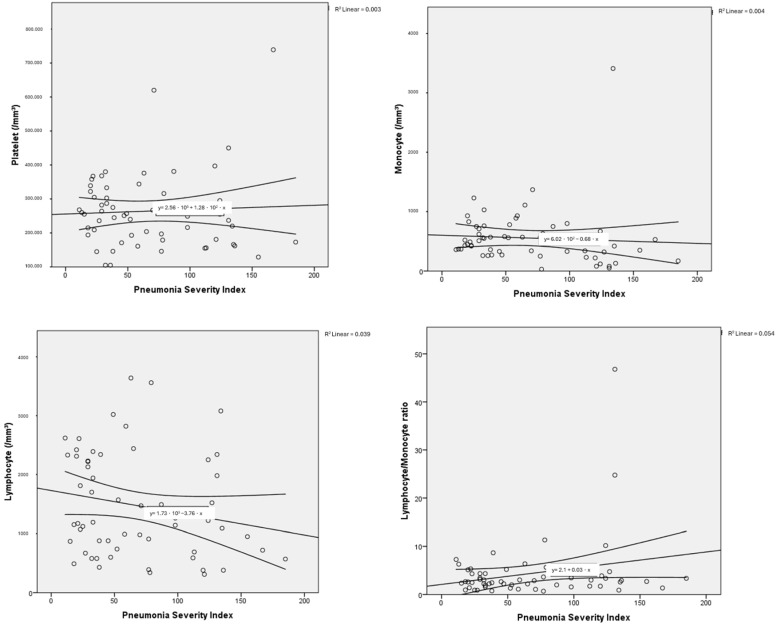
Correlation dot charts between the Pneumonia Severity Index and haematological parameters in viral pneumonia associated with SARS-CoV-2.

The haemogram parameters of patients diagnosed with influenza A/B and SARS-CoV-2 were investigated in terms of their correlation with PSI scores used to classify pneumonia severity and the number of lung lobes with pneumonic infiltration. In this comparison, a partial correlation test was applied to control for the possible confounding effect of age. In particular, a moderate negative correlation was found between haemoglobin and PSI scores, as well as the number of lung lobes with pneumonic infiltration, for both influenza A/B and SARS-CoV-2. Negative low-moderate correlations were found between PSI scores and lymphocyte, platelet, and monocyte counts for influenza A/B (R: −0.402, *p*: 0.005; R: −0.331, *p*: 0.021; R: −0.327, *p*: 0.023, respectively). A positive moderate correlation was found between NLR and PLR with PSI scores (R: 0.527, *p*: <0.001; R: 0.401, *p*: 0.005, respectively). Positive low-moderate correlations were found between PSI scores and leukocyte, neutrophil counts, NLR, LMR, PLR for SARS-CoV-2 (R: 0.392, *p*: 0.003; R: 0.466, *p*: <0.001; R: 0.466, *p*: <0.001; R: 0.278, *p*: 0.036; R: 0.338, *p*: 0.01, respectively). Detailed results are presented in Table 3, along with point correlation graphs in Figure 3 and Figure 4.

## 4. Discussion

In our study, the primary outcome was the seasonal distribution of viral pathogens in 2024 following the COVID-19 pandemic. Influenza A/B was found to be predominantly seen during winter months, consistent with the literature. SARS-CoV-2 was found to be more common during summer months, while Human Rhinovirus was more prominent during summer months but present throughout the year. The seasonal distribution of other viral pathogens could not be determined due to the low number of cases. A six-year retrospective cohort study involving 32,599 pediatric patients was conducted in the literature, and it was found that influenza A/B was predominantly seen during winter months, while Human Rhinovirus was present throughout the year [23,24,25]. A recent review also showed that influenza A/B was more common during winter months, consistent with our study [2]. Other studies have shown that seasonal virus distribution was disrupted during the COVID-19 pandemic but returned to levels similar to previous literature after the pandemic [3,5].

A recent study has shown that SARS-CoV-2 peaked in late September and early October during the 2024–2025 period, similar to our study [24]. However, most studies in the literature have found that SARS-CoV-2 does not exhibit a seasonal distribution and is commonly observed at different times of the year, depending on the emergence of new variants and social factors [4,5]. These studies are mostly based on data collected during the COVID-19 pandemic. There may be several reasons for the detection of SARS-CoV-2, particularly during the summer period, as observed in our study. In the region where this study was conducted, the harvest season for walnuts, a primary source of income, is between August and September. Therefore, seasonal labour migration may have contributed to the prevalence of SARS-CoV-2 during the summer months. The predominance of SARS-CoV-2 during the summer months observed in our study reflects a single-center experience and may not generalize to other regions. However, local public health reports during the same period showed similar seasonal patterns, supporting the validity of our findings. Further studies are needed to determine the seasonal distribution of SARS-CoV-2 after the COVID-19 pandemic. Additionally, it should be noted that the data in our study are not from a general population screening but are based on viral pneumonia pathogens identified in the emergency department.

In studies comparing SARS-CoV-2 and influenza A/B with PSI in terms of pneumonia severity, elevated PSI was found to be associated with disease severity and mortality [19,26,27,28,29]. In our study, PSI class 1 and 2 were detected in 60.3% of SARS-CoV-2-positive patients and 58% of influenza A/B-positive patients, showing similar rates. However, PSI Class 1 was more common in SARS-CoV-2. This may be related to the vaccines administered during the COVID-19 pandemic. However, it has been shown that both viral pneumonias cause viral pneumonias of similar severity after the COVID-19 pandemic. We used the Pneumonia Severity Index (PSI) because it is widely validated and routinely applied in our clinical practice. Alternative scores such as CURB-65 or qSOFA were not systematically recorded in our dataset and therefore could not be assessed retrospectively. Hematological ratios should be regarded as complementary to, rather than substitutes for, standard severity scores. There is limited data on adult populations in this context in the literature, and our study fills this gap.

It has been determined in the literature that haematological parameters change in relation to viral infections [30,31,32]. However, it has been stated that these changes are due to differences in immune mechanisms [7]. In influenza A/B, it has been shown that neutrophil and monocyte infiltration play a role in the inflammatory response and that increased neutrophils and monocytes are associated with disease severity [7]. In another study, it was observed that severe influenza A/B pneumonia was associated with monocytopenia [33,34,35]. In our study, it was found that the monocyte count was statistically significantly lower in patients with influenza A/B who required hospitalisation (*p*: 0.022). Additionally, a low moderate negative correlation was found between PSI and monocyte count (R: −0.327, *p*: 0.023). This may be related to the decrease in monocytes in peripheral blood during the acute phase or an inadequate monocyte response associated with severe disease. Although the correlation coefficient was not high, it should be noted that patients with moderate to severe viral pneumonia were evaluated. This relationship has been demonstrated in a limited number of studies, and our study is consistent with the limited number of studies in the literature, suggesting that monocytopenia may serve as a biomarker for determining the severity of influenza A/B infection.

In our study, the PSI and the number of lung lobes with pneumonic infiltrations were used to determine the severity of influenza A/B-associated disease. Both the increase in PSI and the number of lung lobes with pneumonic infiltrations were negatively correlated with lymphocyte count (R: −0.402, *p*: 0.005; R: −0.290, *p*: 0.046), platelet count (R: −0.331, *p*: 0.021; R: −0.309, *p*: 0.032) and NLR (R: 0.527, *p*: <0.001; R: 0.372, *p*: 0.009). PLR, on the other hand, was found to be moderately correlated only with PSI. In a study, PLR was found to be an important marker for disease severity in viral pneumonia; however, no association was demonstrated between PLR and the number of lung lobes with pneumonic involvement. Lymphocyte count was suggested as a more important marker for indicating the extent of pulmonary infiltration [36,37].

Studies in the literature have reported that NLR and PLR have high sensitivity and specificity in the diagnosis of influenza A/B [21]. It has been demonstrated that lymphocytes and platelets decrease in individuals infected with influenza [21,22]. In a cohort study, lymphocytopenia was shown to play a key role in hospitalisation in patients diagnosed with H1N1 influenza, consistent with our findings [38]. Influenza A/B pneumonia suggests that lymphopenia and increased NLR may play a key role in predicting disease severity and mortality.

In SARS-CoV-2 infections, increases in neutrophil counts and NLR, along with decreases in lymphocyte and haemoglobin counts, have been associated with disease severity [31,32]. Consistent with the literature, our study found that, compared to the control group, patients with SARS-CoV-2 had increased leukocyte, neutrophil, PLR, and NLR levels, and decreased lymphocyte, monocyte, eosinophil, and lymphocyte-monocyte ratio (LMR) levels. An association was observed between the need for hospitalisation and increased neutrophil counts, NLR, and eosinophilia. The association of eosinophilia with disease severity in our study was consistent with the literature [39,40]. Increases in neutrophil count, NLR, LMR, and PLR were found to be significantly associated with the severity of SARS-CoV-2 pneumonia, consistent with the literature [41,42]. These markers may provide diagnostic clues regarding the systemic inflammatory profile of SARS-CoV-2 pneumonia.

Our findings suggest that simple hematological indices such as NLR, LMR, and PLR can provide rapid, low-cost information to guide initial risk stratification in viral pneumonia. While they cannot replace validated biomarkers such as CRP and procalcitonin, they may complement them, especially in settings where advanced assays are not readily available.

The main strength of this study is that it evaluated both combined and separate inflammatory haematological responses in pneumonias caused by different viral agents. While the literature generally consists of comparisons with control groups or studies focused on a single viral agent, this research analysed a wide range of agents in detail. Excluding mild infections and including only cases confirmed by PCR has enabled more reliable assessment of inflammation parameters. The use of the same highly sensitive PCR panel in all patients increased diagnostic consistency; the exclusion of confounding variables such as comorbidities, medication use, smoking, and bacterial infections that could affect the haemogram strengthened internal validity. Diagnostic errors related to emergency department conditions were clearly stated in the methods section, and epidemiological uncertainties related to SARS-CoV-2 were eliminated using data from the post-pandemic period.

This study has several limitations. Due to the case–control design, only associations can be established rather than causality, and the retrospective nature of data collection may have led to missing or inaccurate clinical and laboratory records. Patients with multiple viral detections on PCR were excluded, which may have reduced the epidemiological accuracy of viral distribution. In the busy emergency ward setting, physician-directed patient sampling and high workload may have introduced bias, particularly through underrepresentation of milder cases and the risk of systematic errors. Although sputum culture was requested for all patients, inappropriate or contaminated results were excluded, and a negative culture does not fully rule out bacterial pneumonia. While hematological parameters are practical and widely used, they are nonspecific and may be influenced by other systemic or physiological factors. More specific biomarkers such as procalcitonin, CRP, and IL-6 were not consistently available and therefore could not be included, which may restrict the generalizability of our findings compared with studies using multi-marker panels. Despite careful exclusion of confounding factors, residual confounding from unreported variables such as immune status, subclinical disease, or co-infections cannot be entirely ruled out. Finally, although the study was adequately powered for the most common viral etiologies (SARS-CoV-2 and influenza A/B), the relatively small sample size and single-center design limit statistical power for subgroup analyses and generalizability to broader populations. These limitations were mitigated by the use of a homogeneous diagnostic protocol, strict exclusion criteria, and post-pandemic sample selection.

In conclusion, the study demonstrates that inflammatory haematological responses to different viral pathogens can be distinguished, providing a low-cost and accessible tool for diagnosis. The focus on moderate-to-severe pneumonia cases and the demonstration of seasonal distribution have strengthened the clinical relevance of the observed changes and contributed to the prediction of epidemic periods. The findings suggest that haematological parameters may play a supportive role in areas such as rapid decision-making in emergency departments, determining disease severity, and reducing unnecessary antibiotic use.

## Figures and Tables

**Table 1:** Characteristics of the Study and Control Groups.

	Study Group				Control Group			
	n	%	Mean ± Standard Deviation	%95 CI	n	%	Mean ± Standard Deviation	%95 CI
Age			50.40 ± 19.36	46.12–52.68			51.19 ± 21.36	47.30–54.98
Gender								
Female	70	54.3			73	56.6		
Male	59	45.7			56	43.4		

Influenza A/B (Age)			53.98 ± 21.88	48.0–59.84				
SARS-CoV2 (Age)			51.45 ± 20.66	46.98–56.79				
Seasonal Disturbance of Viral Pathogens								
		Influenza A/B (n: 49% 38)	SARS-CoV2 (n: 58% 45)	Human Rhinovirus (n: 14% 10,9)		RSV (n: 3% 2,3)	Adenovirus (n: 4% 3,1)	Parainfluenza Virus (n: 1% 0,7)
January–February–March		7 (%14.3)	4 (%6.9)	1 (%7.2)		2 (%66.7)	1 (%25)	1 (%100)
April–May–June		9 (%18.4)	5 (%8.6)	3 (%21.4)		1 (%33.3)	2 (%50)	0
July–August–September		0	41 (%70.7)	5 (%35.7)		0	0	0
October–November–December		33 (%67.3)	8 (%13.8)	5 (%35.7)		0	1 (%25)	0
RxC test: Fisher’s Exact Test *p* value: <0.001								
Treatment Decision								
Outpatient		35 (%71.4)	44 (%75.9)	14 (%100)		2 (%66)	3 (%75)	1 (%100)
Hospitalisation		14 (%28.6)	14 (%24.1)	0		1 (%33.3)	1 (%25)	0
Pneumonia Severity Index (PSI)								
PSI—Class 1		11 (22.4%)	26 (44.8%)	7 (50%)		1 (33%)	4 (100%)	0
PSI—Class 2		15 (30.6%)	9 (15.5%)	3 (21.4%)		1 (33%)	0	0
PSI—Class 3		8 (16.3%)	6 (10.3%)	4 (28.6%)		0	0	1 (100%)
PSI—Class 4		6 (12.2%)	12 (20.7%)	0		0	0	0
PSI—Class 5		9 (18.3%)	5 (8.6%)	0		1 (33%)	0	0
RxC test: Pearson Chi-square *p* value: 0.035; RSV: Respiratory Syncytial Virus								

**Table 2:** Comparison of haematological parameters of SARS-CoV-2 and Influenza A/B with the control group.

	Control Group (n: 129)	SARS-CoV2 (n: 58)	Influenza A/B (n: 49)	*p* Value
Hemoglobin (g/dL)	14.56 ± 1.72	12.86 ± 1.86	12.92 ± 2.16	<0.001 _⸸_ <0.001 _⸶_
Leukocyte (/mm^3^)	7449.3 ± 1492.87	8863.7 ± 6225.66	7022 ± 2717.3	0.326 _⸸_ 0.019 _⸶_
Neutrophil (/mm^3^)	4324.03 ± 1290,54	6680.15 ± 5666.82	5423.8 ± 2689.8	<0.001 _⸸_ 0.016 _⸶_
Lymphocyte (/mm^3^)	2294.65 ± 699,88	1475.1 ± 883.67	1018.7 ± 678.6	<0.001 _⸸_ <0.001 _⸶_
Platelet (/mm^3^)	261,937.98 ± 63,023.52	264,310.34 ± 113,142	217,530 ± 77,739	0.489 _⸸_ <0.001 _⸶_
Monocyte (/mm^3^)	581.0 ± 154.40	556.03 ± 481.59	470.4 ± 251.0	0.012 _⸸_ <0.001 _⸶_
Eosinophil (/mm^3^)	201.86 ± 171.45	96.72 ± 140.3	76.12 ± 131.16	<0.001 _⸸_ <0.001 _⸶_
NLR	2.15 ± 1.42	6.29 ± 6.35	9.36 ± 10.10	<0.001 _⸸_ <0.001 _⸶_
LMR	4.16 ± 1.56	4.36 ± 6.71	2.46 ± 1.62	<0.001 _⸸_ <0.001 _⸶_
PLR	126.86 ± 59.45	249.78 ± 198.68	306.0 ± 200.17	<0.001 _⸸_ <0.001 _⸶_

Student *t*-test. ⸸: Comparison of haematological parameters of SARS-CoV-2 with the control group. ⸶: Comparison of haematological parameters of Influenza A/B with the control group. NLR: Neutrophil/Lymphocyte ratio, LMR: Lymphocyte/Monocyte ratio, PLR: Platelet/Lymphocyte ratio.

**Table 3:** Correlation between SARS-CoV2 and Influenza A/B Haematological Parameters and Pneumonia Severity Index.

**Influenza A/B**		
	**Pneumonia Severity Index**	**Number of Lobes Showing Pneumonic İnfiltration**
Hemoglobin (g/dL)	R: −0.502, *p*: 0.00	R: −0.552, *p*: 0.00
Leukocyte (/mm^3^)	R: −0.031, *p*: 0.833	R: −0.098, *p*: 0.506
Neutrophil (/mm^3^)	R: 0.108, *p*: 0.465	R: −0.021, *p*: 0.887
Lymphocyte (/mm^3^)	R: −0.402, *p*: 0.005	R: −0.290, *p*: 0.046
Platelet (/mm^3^)	R: −0.331, *p*: 0.021	R: −0.309, *p*: 0.032
Monocyte (/mm^3^)	R: −0.327, *p*: 0.023	R: −0.193, *p*: 0.188
Eosinophil (/mm^3^)	R: 0.075, *p*: 0.611	R: 0.348, *p*: 0.015
NLR	R: 0.527, *p*: <0.001	R: 0.372, *p*: 0.009
LMR	R: −0.232, *p*: 0.112	R: −0.179, *p*: 0.224
PLR	R: 0.401, *p*: 0.005	R: 0.234, *p*: 0.109
**SARSCoV2**		
	**Pneumonia Severity Index**	**Number of Lobes Showing Pneumonic İnfiltration**
Hemoglobin (g/dL)	R: 0.218, *p*: 0.104	R: 0.232, *p*: 0.082
Leukocyte (/mm^3^)	R: 0.392, *p*: 0.003	R: 0.729, *p*: <0.001
Neutrophil (/mm^3^)	R: 0.466, *p*: <0.001	R: 0.765, *p*: <0.001
Lymphocyte (/mm^3^)	R: −0.162, *p*: 0.229	R: 0.102, *p*: 0.450
Platelet (/mm^3^)	R: 0.183, *p*: 0.173	R: 0.314, *p*: 0.017
Monocyte (/mm^3^)	R: −0.137, *p*: 0.308	R: −0.254, *p*: 0.057
Eosinophil (/mm^3^)	R: −0.133, *p*: 0.324	R: −0.172, *p*: 0.199
NLR	R: 0.466, *p*: <0.001	R: 0.450, *p*: <0.001
LMR	R: 0.278, *p*: 0.036	R: 0.242, *p*: 0.070
PLR	R: 0.338, *p*: 0.01	R: 0.127, *p*: 0.347

A partial correlation test was performed with age controlled. R: correlation coefficient, *p*: *p* value. NLR: Neutrophil/Lymphocyte ratio, LMR: Lymphocyte/Monocyte ratio, PLR: Platelet/Lymphocyte ratio.

## Data Availability

After obtaining ethical committee approval, permission was obtained from the Ministry of Health to conduct a retrospective data search. In accordance with the Ministry’s policy, data sharing is subject to permission. Therefore, permission must be obtained from the Ministry of Health for data sharing.

## Abbreviations

ARDS	Acute Respiratory Distress Syndrome
HIMS	Hospital Information Management System
H	Hypotheses
LMR	Lymphocyte/Monocyte Ratio
NLR	Neutrophil/Lymphocyte Ratio
PLR	Platelet/Lymphocyte Ratio
PSI	Pneumonia Severity Index
RSV	Respiratory Syncytial Virus
